# Mitigating Neural Habituation in Insect Bio-Bots: A Dual-Timescale Adaptive Control Approach

**DOI:** 10.3390/biomimetics11010013

**Published:** 2025-12-27

**Authors:** Le Minh Triet, Nguyen Truong Thinh

**Affiliations:** Institute of Intelligent and Interactive Technologies, University of Economics Ho Chi Minh City—UEH, Ho Chi Minh City 70000, Vietnam; lmt30062003@gmail.com

**Keywords:** Vietnam’s cyborg, ANFIS, search and rescue cyborg, bio-cybernetics, fuzzy logic, neural stimulation, cockroach navigation, neuroengineering

## Abstract

Bio-cybernetic organisms combine biological locomotion with electronic control but face significant challenges regarding individual variability and stimulus habituation. This study introduces an Adaptive Neuro-Fuzzy Inference System (ANFIS) designed to dynamically calibrate to individual *Gromphadorhina portentosa* specimens. Using a miniaturized neural controller, we compared ANFIS’s performance against natural behavior and non-adaptive control methods. Results demonstrate ANFIS’s superiority: obstacle navigation efficiency reached 81% (compared to 42% for non-adaptive methods), and effective behavioral modulation was sustained for 47 min (versus 26 min). Furthermore, the system achieved 73% target acquisition in complex terrain and maintained stimulus responsiveness 3.5-fold longer through sophisticated habituation compensation. Biocompatibility assessments confirmed interface functionality over 14-day periods. This research establishes foundational benchmarks for arthropod bio-cybernetics, demonstrating that adaptive neuro-fuzzy architectures significantly outperform conventional methods, enabling robust bio-hybrid platforms suitable for confined-space search-and-rescue operations.

## 1. Introduction

The convergence of biological systems with advanced computational controls represents one of the most compelling frontiers in contemporary robotics, particularly for humanitarian applications in disaster response. In the aftermath of catastrophic seismic events, the search for survivors is often impeded by unstable rubble, collapsed infrastructure, and confined voids that remain inaccessible to canine teams or traditional rigid robots. Bio-cybernetic systems—specifically those utilizing the Madagascar hissing cockroach (*Gromphadorhina portentosa*)—present an unprecedented opportunity to address this critical gap. By harnessing the insect’s evolutionary adaptations for agile locomotion in dark, unstructured environments, and augmenting them with electronic navigation systems, we can create autonomous agents capable of penetrating deep into earthquake debris to locate survivors. However, the transition from biological potential to reliable search-and-rescue asset is obstructed by a fundamental challenge: developing control architectures capable of effectively managing the inherent complexity, uncertainty, and nonlinearity that characterize living organisms.

Early implementations of bio-cybernetic control relied on linear strategies adapted from traditional robotics, but these quickly revealed the limitations of applying rigid engineering logic to biological systems. The pioneering work establishing proof-of-concept neural stimulation [1] employed basic switching mechanisms that provided only rudimentary directional influence. When researchers attempted to refine this with proportional control [2], the systems suffered a severe 60% performance degradation within ten minutes. This failure was primarily due to neural habituation—a biological mechanism where the insect’s nervous system desensitizes to repetitive electrical signals—which fixed-gain linear methods could not mitigate. Similarly, linear state feedback control [3] encountered high failure rates across 40% of test specimens because it assumed system linearity and time-invariance, conditions that are categorically violated by the dynamic physiology of individual insects.

Subsequent efforts to bridge this gap through modeling and optimization faced comparable hurdles. Neural network models designed for model predictive control [4] struggled with model-plant mismatches, achieving success rates below 45% due to the impossibility of accurately simulating a nervous system with over 100,000 interconnected neurons. Biomechanical modeling proved equally limited, failing to capture the stochastic nature of insect movement. While hybrid approaches combining PID with basic fuzzy logic [5] showed initial promise, they lacked the learning capabilities to maintain control over extended periods, leading to 35% effectiveness degradation. Furthermore, genetic algorithm optimization [6] functioned well in offline simulations but failed to provide the real-time adaptation necessary to respond to the rapid biological changes occurring during active navigation.

Even sophisticated nonlinear control methodologies have demonstrated fundamental inadequacies when applied to the chaotic environment of a bio-bot. Sliding mode control [7] while robust, induced high-frequency chattering that caused tissue damage in 25% of subjects. Adaptive control implementations [8] often destabilized into oscillatory behavior when inherent biological noise overwhelmed parameter identification algorithms. Deep reinforcement learning [9], a leading candidate for autonomous navigation, experienced performance collapse after just 20 min because standard reward functions failed to account for the complex dynamics of neural habituation. Other advanced techniques, including support vector machines [10], feedback linearization [11], H-infinity control [12], neural model predictive control [13], and fractional-order PID [14], all struggled to balance computational efficiency with the need to adapt to rapid parameter drift and individual biological variability.

The challenge extends to the coordination of these agents for swarm-based search-and-rescue. Distributed control approaches, such as multi-agent consensus [15] and event-triggered control [16], frequently resulted in unstable behaviors due to inter-channel coupling effects. Optimization techniques like particle swarm optimization [17] proved too slow for real-time biological response, requiring convergence times that exceeded the millisecond-scale reflexes of the insects. These failures underscore a universal truth in bio-engineering: fixed or slowly adapting controllers are insufficient for biological systems. Sliding mode control is robust to bounded uncertainties but may induce chattering and typically requires reliable bounds for smooth, safe stimulation. Deep reinforcement learning can model highly nonlinear dynamics but is data- and compute-intensive and complicates safety-constrained training. We therefore adopt ANFIS for its interpretable rule base, lightweight inference, and online adaptability under inter-individual variability and habituation.

However, evidence from parallel fields suggests a solution. Research in neural prosthetics [18,19] and functional electrical stimulation [20,21] has consistently shown that adaptive, nonlinear controllers significantly outperform linear models by learning individual physiological patterns. Similar successes in heart rate regulation [22] pharmaceutical fermentation [23,24], insulin delivery [25], and greenhouse crop management confirm that adaptive intelligent methodologies can successfully manage biological uncertainty where conventional controls fail. The main contribution is a dual-timescale adaptive ANFIS framework for habituation-aware bio-bot navigation: fast RLS updates of consequent parameters track short-term response drift, while slower gradient/LMA refinement of membership functions captures long-term, individual-specific adaptation.

Building on our previous work identifying the limitations of reinforcement learning regarding individual variability [26], this study introduces an Adaptive Neuro-Fuzzy Inference System (ANFIS) designed specifically for the rigors of confined-space search and rescue. Unlike prior approaches, ANFIS continuously refines control parameters based on real-time responses, effectively creating a unique control profile for each cyborg that evolves as the insect fatigues or habituates. By integrating this real-time learning with interpretable fuzzy logic, we aim to provide the sustained, reliable navigation capability required to deploy bio-bots into the unpredictable depths of earthquake ruins.

The remainder of this paper is organized as follows: Section 2 details our methodological approach and experimental setup. Section 3 presents the ANFIS-based control system design. Section 4 reports the experimental results, and Section 5 concludes with insights for future research and ethical considerations.

## 2. Materials and Methods

*G. portentosa* was selected as the biological platform due to its robust physiology and well-documented neuroanatomy. To minimize variability, adult specimens were sourced from a controlled colony maintained at 27 ± 1 °C and 60 ± 5% humidity. Subjects were strictly selected based on age (45 ± 5 days), size, and weight (15–22 g) [27], ensuring consistent locomotor activity. Prior to interface attachment, specimens underwent brief CO_2_ anesthesia (2 L/min for 30 s). The dorsal thoracic exoskeleton was then gently abraded and treated with a custom biocompatible adhesive to ensure secure, long-term fixation of the electronic backpack.

### 2.1. Bio-Electronic Interface Design and Experimental Setup

**Figure 1:**
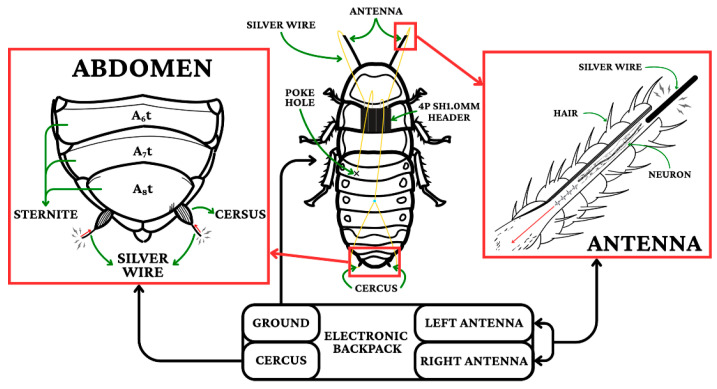
Electrode placement diagram.

The interface utilized electrochemically treated 50 μm silver wire electrodes with 125 μm exposed tips to minimize impedance. Antennal electrodes were implanted 0.8 mm from the base at a depth of 2.1 mm to interface with the antennal nerve, while cercal electrodes (75 μm wire) were positioned with a 0.5 mm inter-electrode distance to facilitate differential stimulation, the lines are showed as yellow (Figure 1). The onboard electronics were engineered with strict size and weight constraints to preserve natural locomotion.

**Figure 2:**
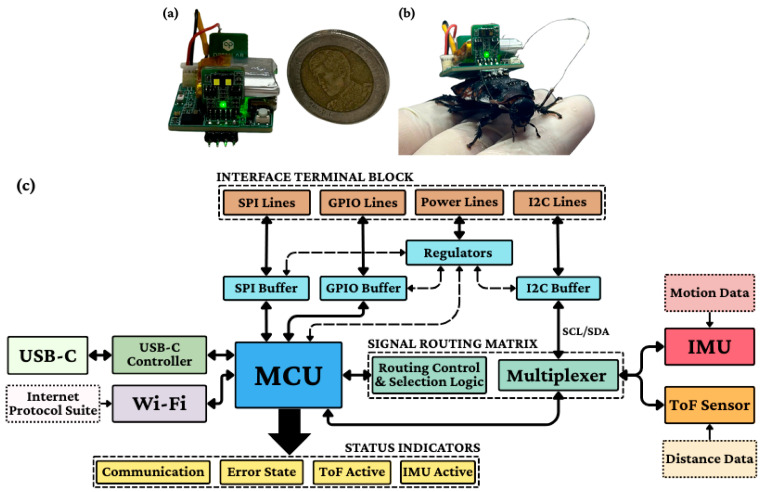
Actual size of electronic backpack (**a**), schematic illustration of the bio-bot backpack structure and body alignment (**b**) and the hardware architecture (**c**) of the backpack.

**Figure 3:**
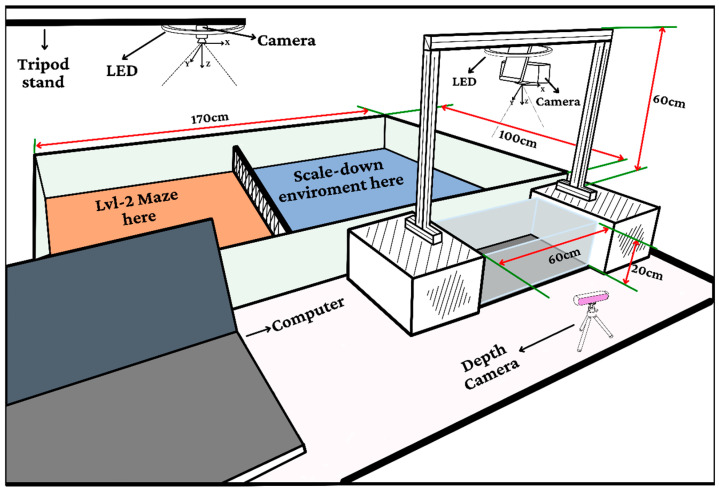
Experimental arena configurations.

The validated control system utilizes a miniaturized electronics package weighing 4.85 g (2.9 × 2.6 cm), depicted in Figure 2a and shown mounted on the host in Figure 2b. The hardware architecture (Figure 2c) comprises two modules: Module A features an ESP32-S3 microcontroller (Espressif Systems, Shanghai, China)and BNO055 9-DOF IMU (Bosch Sensortec, Reutlingen, Germany) for processing and motion sensing, while Module B incorporates VL53L1X Time-of-Flight sensors (STMicroelectronics, Geneva, Switzerland) for precision ranging up to 4 m (±3% accuracy). These modules communicate via 400 kHz I2C, powered by a hybrid 100 mAh Li-Po battery system. The 170 cm experimental arena (Figure 3) includes maze and scale-down testing zones monitored by overhead, secondary, and depth cameras, enabling real-time behavioral analysis through a centralized computer interface.

### 2.2. Behavioral Characterization and Modeling

To develop the ANFIS controller, we compiled a comprehensive dataset using 5 distinct specimens over four weeks, yielding approximately 750,000 time-stamped state-action points. Ground-truth control actions were established using a “Wizard-of-Oz” methodology, where an expert operator manually guided the subjects to capture nuanced control strategies. The resulting data underwent rigorous preprocessing, including synchronization, normalization, and Kalman filtering, under standardized environmental conditions (25 ± 1 °C, 15 lux). Analysis revealed two critical habituation mechanisms: short-term recovery after 6–12 episodes and progressive long-term adaptation after 45 min. To address this, we implemented a modified Wiener cascade architecture to model response dynamics. The observed 45% inter-specimen variability in response thresholds fundamentally justified the adoption of our adaptive control architecture over fixed-parameter systems (Table 1).

### 2.3. Comparative Control Methodology

To rigorously evaluate the ANFIS controller, we implemented a fixed-parameter PID Fuzzy-Hybrid system representing conventional bio-cybernetic approaches. This methodology combines linear PID strategies for foundational movement with static fuzzy logic rules based on predetermined stimulation parameters. Unlike the adaptive ANFIS, this fixed-gain framework lacks learning capabilities and assumes system linearity and time-invariance—assumptions categorically violated by biological organisms. Consequently, this non-adaptive approach failed to accommodate stochastic variability, neural excitability drift, and habituation dynamics, resulting in significant performance degradation and rendering the system ineffective for sustained operations.

Table 2 summarizes trade-offs among advanced nonlinear controllers. SMC offers robustness but its discontinuous structure is difficult to smooth without added design complexity; DRL is flexible but training-heavy and safety-limited in many bio-bot settings. ANFIS provides efficient, interpretable inference with low-data online adaptation, making it practical for resource-limited habituation-aware control.

## 3. Architecture of Adaptive Neuro-Fuzzy Inference System

### 3.1. ANFIS-Based Control System Design

**Figure 4:**
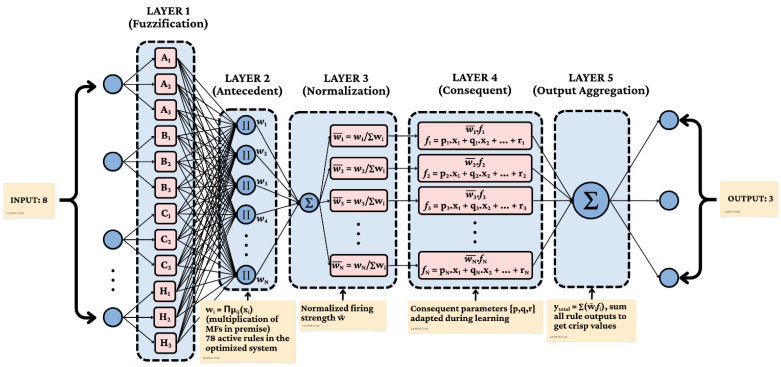
ANFIS architecture for bio-cybernetic control.

The ANFIS architecture represents a sophisticated fusion of neural network learning capabilities with fuzzy logic reasoning, creating a hybrid system particularly well-suited to bio-cybernetic control applications. Our implementation is founded on a five-layer network structure that systematically transforms crisp input values into appropriate stimulation parameters through interconnected processing layers. Each layer serves a specific computational purpose within the overall architecture, as illustrated in Figure 4 and ANFIS model configuration is summarized in Table 3.

Active rules: typically ≈105 (bounded 80–150 via online grow/prune). The first layer performs input fuzzification, where each input value is converted into a membership degree for a relevant fuzzy set. This is achieved using a Type-1 generalized bell-shaped membership function (gbellmf), defined by the equation:

(1) $$\mu (x)=\left\{\begin{array}{ll} \mu_{1}(x) & \mathrm{if}\;x\leq c\\ \mu_{2}(x) & \mathrm{if}\;x>c \end{array}\right.$$

where

(2) $$\mu_{1}(x)=\exp\left(-\frac{{\left(x-c\right)}^{2}}{2\sigma_{1}^{2}}\right)$$

(3) $$\mu_{2}(x)=\exp\left(-\frac{{\left(x-c\right)}^{2}}{2\sigma_{2}^{2}}\right)$$

Here, a controls width, b controls steepness, and c is controls the center (generalized bell MF). A common and effective alternative is the Gaussian membership function, which uses a center *c* and standard deviation *σ* to define its classic bell shape via (2). For square M, diag(M) extracts the diagonal as a vector; Diag(v) constructs a diagonal matrix with diagonal v (3). The use of any of these functions ensures that the premise parameters can be reliably updated during the ANFIS training process. It is calculated as the algebraic product (T-norm) of the input membership values corresponding to that rule:

(4) $$O_{k}^{\left(2\right)}=w_{k}=\prod_{i=1}^{n}\mu_{A_{i,k}}(x_{i})$$

This multiplicative aggregation implements the logical AND operation in the antecedent of fuzzy rules, producing higher firing strengths when all conditions are strongly satisfied. The third layer performs rule strength normalization, calculating the relative importance of each rule within the active rule set:

(5) $${\bar{w}}_{i}=\frac{w_{i}}{\sum_{j}w_{j}}$$

where ${\bar{w}}_{i}$ represents the normalized firing strength of the $i^{th}$ rule, ensuring that the sum of all normalized rule strengths equals unity regardless of the absolute magnitude of firing strengths. The output of the *i*-th rule, *f_i_*, is a linear polynomial of the input variables, which is characteristic of a first-order Sugeno model. The output is calculated as:

(6) $$f_{i}={\bar{w}}_{i}\left(p_{i0}+p_{i1}x_{1}+p_{i2}x_{2}+\ldots +p_{in}x_{n}\right)$$

Here, ${\bar{w}}_{i}$ is the normalized firing strength from Layer 3, and {*p_i_*_0_, *p_i_*_1_, *…*,*p_in_*} are the consequent parameters for the *i*-th rule, which are updated during the learning process. The fifth layer performs output aggregation through summation of individual rule contributions:

(7) $$y=\sum_{i}{\bar{w}}_{i}f_{i}$$

This weighted sum combines the recommendations from all relevant rules according to their normalized firing strengths, producing the final crisp output value that determines stimulation parameters.

### 3.2. Backpropagation and Least Squares

During the forward pass, the consequent parameters *θ* are updated using Recursive Least Squares (RLS) with a forgetting factor *λ* (set to 0.995). The update equations are:

(8) $$K_{t+1}=\frac{P_{t}a_{t+1}}{\lambda +a_{t+1}^{T}P_{t}a_{t+1}}$$

(9) $$\theta_{t+1}=\theta_{t}+K_{t+1}\left(y_{t+1}-a_{t+1}^{T}\theta_{t}\right)$$

(10) $$P_{t+1}=\frac{1}{\lambda }\left(P_{t}-K_{t+1}a_{t+1}^{T}P_{t}\right)$$

where ***a****_t_* is the vector of normalized inputs, **P** is the covariance matrix, **θ** is the vector of consequent parameters, and *y* is the target output. In the backward pass, consequent parameters remain fixed while antecedent parameters are adjusted using GD to minimize output error, the Levenberg–Marquardt Algorithm (LMA) updates the parameter vector **α** by solving for the update step **Δα**:

(11) $$\left(J^{T}J+\lambda \mathrm{diag}(J^{T}J)\right)\Delta \alpha =J^{T}e$$

(12) $$\alpha_{k+1}=\alpha_{k}+\Delta \alpha$$

where **α** is the vector of all premise parameters to be optimized, **J** is the Jacobian matrix of the error vector with respect to the parameters, *λ* is a damping factor that is adaptively adjusted at each iteration, **e** is the vector of errors between the ANFIS output and the target values. Backpropagation is implemented with momentum terms to accelerate convergence and avoid local minima.

### 3.3. ANFIS Structure Implementation

The controller processes eight primary input variables capturing cockroach state and environmental context, Table 4 shows all the selected input variables for the ANFIS controller and their respective fuzzy sets. To capture the distinct temporal patterns of habituation, the dual-state habituation model is augmented with a state-dependent increment factor and a stochastic term based on Wiener process increments (*dW_t_*):

(13) $$dH_{s}(t)=\left(-D_{s}H_{s}(t)+I_{s}(t)\cdot S(t)\right)dt+\sigma_{s}dW_{s}(t)$$

(14) $$dH_{l}(t)=\left(-D_{l}H_{l}(t)+I_{l}\cdot H_{s}(t)\right)dt+\sigma_{l}dW_{l}(t)$$

where the short-term habituation increment *I_s_* depends on the cockroach’s linear velocity *v*(*t*).

(15) $$I_{s}(t)=I_{s0}\exp(-k_{v}\cdot v(t))$$

where t is time and dt is the update interval. H(t)∈[0,1] is the habituation state with short- and long-term components $H_{s}(t)$ and $H_{l}(t)$. v(t) is normalized linear velocity, $I_{s}$ is a velocity-dependent short-term increment term, and $dW_{t}$ is a Wiener increment modeling stochastic biological variability; remaining coefficients set drift, coupling, and noise magnitudes. This differential equation form models habituation as a continuous-time process. The term *I_s_*(*t*) implies that habituation occurs more slowly when the animal is moving faster. The terms *σ_s_* and *σ_l_* scale the biological noise.

### 3.4. Learning and Adaptation Mechanisms

The stimulation parameter *P*(*t*) is modulated using a linear chirp signal, where the instantaneous frequency changes based on the habituation level H(*t*):

(16) $$P(t)=P_{base}(t)\cdot \left[1+A(H(t))\cdot \sin\left(2\pi \int_{0}^{t}f(\tau )d\tau \right)\right]$$

where

(17) $$f(t)=f_{start}+k_{f}\cdot H(t)\cdot t$$

This ensures that as habituation increases, the stimulation becomes not only stronger but also more varied in its temporal pattern, preventing the neural system from adapting. These intervals are calibrated to the observed recovery characteristics of each specimen, maximizing recovery opportunity while maintaining navigational performance:

(18) $$\mathrm{IBI}(t)={\mathrm{IBI}}_{\max}-({\mathrm{IBI}}_{\max}-{\mathrm{IBI}}_{\min})\cdot e^{-k\cdot H(t)}$$

where IBI_min_ and IBI_max_ are the minimum and maximum allowable intervals, and *k* is a scaling factor that determines how aggressively the rest period increases with the estimated habituation level. Algorithm 1 outlines the core computational sequence employed by the ANFIS controller. The process initiates by generating or initializing the premise parameters *α* and consequent parameters *θ*. Subsequently, the habituation states *H_s_* and *H_l_* are calculated by integrating their respective differential equations, drawing from the established habituation model. Following this, the forward pass of the ANFIS architecture commences: the membership degree *μ*(*x_t_*) for all inputs is determined using the generalized bell-shaped membership functions as defined in Equations (1)–(3). The firing strength *w_k_* for each fuzzy rule is then computed as the algebraic product of these membership degrees, as specified in Equation (4). These firing strengths are subsequently normalized using Equation (5) to establish the relative importance of each rule. The consequent output *f_i_* for each rule is then generated using a nonlinear sinusoidal model, as presented in Equation (6). Finally, the total ANFIS output *y_t_* is constructed by aggregating these consequent outputs through summation, as per Equation (7). Upon generating the ANFIS output, the algorithm proceeds to produce the modulated stimulation command *P*(*t*) and inter-burst interval IBI(t) by applying Equations (16)–(18), which are crucial for habituation compensation. For continuous adaptation, the algorithm then enters the learning phase: the consequent parameters *θ_new_* are updated using the RLS algorithm, employing Equations (8)–(10). Concurrently, the premise parameters *α_new_* are adjusted by applying the LMA to minimize the output error, as described by Equations (11) and (12). The controller utilizes a powerful hybrid learning algorithm where the consequent parameters are updated in the forward pass using RLS, while the premise parameters are optimized in the backward pass using the LMA to minimize the output error. We implement two coupled update rates: per-step RLS for consequent parameters (with forgetting factor λ) and lower-rate, conservative optimization for membership parameters, improving stability under non-stationarity.
**Algorithm 1: Find the output layer****Require:** Input state vector x_t_ ∈ ℝ^8^ at time *t*, and a training dataset D = {(x_m_, y_m_)} for m = 1,…,M training samples, where y_m_ ∈ ℝ^3^ is the target output. *J* is the number of fuzzy rules. **1:****Generate:** Premise parameters α and consequent parameters *θ* randomly or based on initial knowledge.**2:****Calculate:** The habituation states H_s_ and H_l_ by integrating their respective differential equations using (13)–(15).**3:****Calculate:** The membership degree μ(x_t_) for all inputs using the fuzzy membership functions defined in (1)–(3).**4:****Calculate:** The firing strength *w_k_* for each of the *J* rules using the algebraic product T-norm, as defined in (4).**5:****Calculate:** The normalized firing strength ${\bar{w}}_{i}$ for each rule using (5).**6:****Calculate:** The consequent output *f_i_* for each rule using the nonlinear sinusoidal model in (6).**7:****Construct:** The final ANFIS output y_t_ ∈ ℝ^3^ by aggregating the consequent outputs, as per (7).**8:****Generate:** The modulated stimulation command P(t) and inter-burst interval IBI(t) using (16)–(18).**9:****Find:** The updated consequent parameters θ_new_ by applying the RLS algorithm to a training sample (x_m_, y_m_) using (8)–(10).**10:****Find:** The updated premise parameters α_new_ by applying the LMA to the error *e* using (11) and (12).

### 3.5. Control System Integration

**Figure 5:**
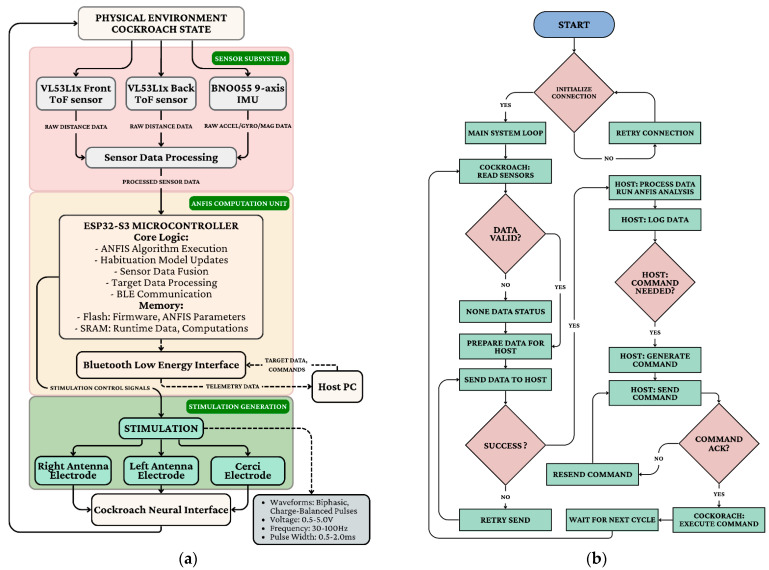
Contrasts host-assisted training/monitoring (prototype) with MCU-only inference using fast consequent adaptation; the video stream is included for evaluation only. (**a**) ANFIS controller hardware implementation; (**b**) Main system loop: read sensors → estimate state → update/check policy → (optional) stimulate → log/communicate → repeat. If no host command/update is needed, the system continues the loop using the current policy (or a no-stim action under the deviation threshold).

To operate within strict miniaturization constraints, the hardware employs specialized voltage-controlled current source circuits that deliver charge-balanced stimuli while adjusting for tissue impedance. The system architecture (Figure 5a) relies on an ESP32-S3 microcontroller to fuse data from time-of-flight distance sensors and a BNO055 9-axis IMU. Acting as the bio-bot’s “brain,” this unit communicates via Bluetooth Low Energy with a Host PC. The closed-loop operational workflow (Figure 5b) offloads computationally intensive ANFIS analysis to the host: the cockroach transmits telemetry, and the host generates corrective stimulation commands only when instinctual movements deviate from the target path, ensuring robust, goal-directed navigation.

**Figure 6:**
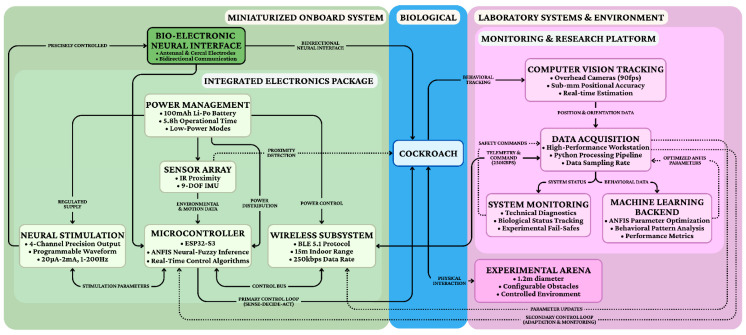
System architecture overview.

Memory access optimization addressed the significant performance impact of memory operations on the target architecture. The computational sequence was reorganized to maximize data locality and minimize cache misses, with frequently accessed parameters stored in dedicated memory regions. The integration of these multiple feedback loops creates a resilient control architecture capable of adapting to changing conditions across different timescales. The primary loop (Figure 6) maintains responsive moment-to-moment control, while the secondary loops continuously refine the control strategy to optimize long-term performance and sustainability. The closed-loop (Figure 5b) implementation successfully addressed the major challenges identified for bio-cybernetic cockroach control, demonstrating robust navigation capabilities that adapted to individual characteristics, compensated for habituation effects, and maintained effectiveness across diverse environmental conditions.

## 4. Results

**Figure 7:**
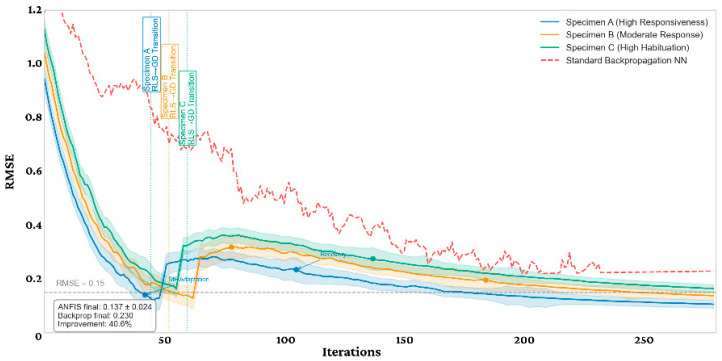
ANFIS learning convergence curve.

This section presents the technical performance metrics of ANFIS controller, focusing on its learning capabilities and computational efficiency. The ANFIS controller demonstrated exceptional learning convergence characteristics across the training phase. Figure 7 illustrates this convergence behavior across multiple specimen training sessions. The experimental results demonstrate that the proposed ANFIS-based control system significantly outperforms standard backpropagation neural networks for bio-cybernetic cockroach control across all test specimens. As illustrated in Figure 7, the ANFIS learning convergence curves exhibit consistently lower error rates, with a final mean RMSE of 0.13 ± 0.02 compared to backpropagation’s 0.23, representing a 43.5% improvement. Specimen A (high responsiveness) showed the fastest convergence at epoch 92 and achieved the lowest final error (0.11), while Specimen C (high habituation) required more extensive training due to its challenging biological characteristics. The hybrid learning approach contributing 62–75% of the total error reduction in significantly fewer epochs. Statistical analysis confirms these findings are highly significant (*p* < 0.001, Cohen’s *d* = 3.74). Importantly, the ANFIS model demonstrated superior adaptability to individual specimen characteristics, with velocity responsiveness and habituation rate emerging as the most influential biological factors affecting control performance. RLS → GD denotes the dual-timescale schedule (fast RLS consequent updates with slow GD/LMA membership refinement). We relabeled this as ‘Fast (RLS) update/Slow (GD) refinement’ to avoid implying a hard runtime mode switch.

**Figure 8:**
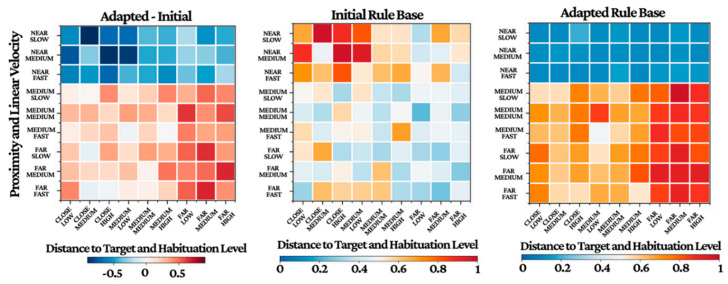
ANFIS rule base adaptation.

**Figure 9:**
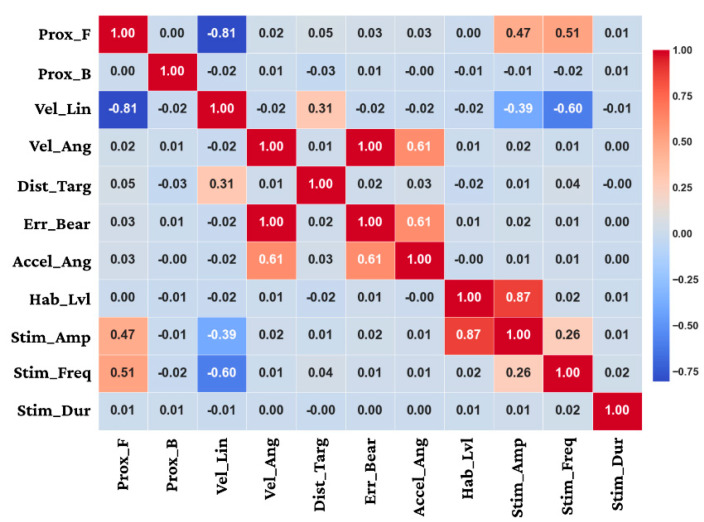
Pearson correlation matrix (−1 to +1) of normalized trial variables. Abbreviations: Prox_F/Prox_B (ToF proximity), Vel_Lin, Vel_Ang, Dist_Targ, Err_Bear (deg), Accel_Ang, Hab_Lvl.

These results validate the effectiveness of fuzzy logic-based approaches for handling the Inherent variability and non-linearity in biological systems, particularly for real-time control applications requiring robust performance despite biological variability. The adaptation of membership function parameters followed distinct patterns for different input variables. Table 5 summarizes the initial and optimized membership function parameters for key input variables. The rule base evolved significantly during both offline and online learning phases. Initial rule structures based on expert knowledge were progressively refined through adaptation to individual specimen characteristics, Figure 8 provides a visualization of this evolution. Besides that, Figure 9 presents the Pearson correlation matrix computed from the experimental data, quantifying the linear relationships between the sensory-state input variables and the corresponding neurostimulation control outputs generated by the ANFIS. The matrix provides statistical validation of the controller’s underlying logic. A very strong positive correlation (r = 0.87) is observed between the estimated habituation level and the stimulus amplitude, serving as direct evidence for the system’s adaptive habituation-compensation mechanism. Furthermore, the core directional control strategy is confirmed by the strong positive correlation (r = 1.00) between the target bearing error and the resulting angular velocity. Essential safety and maneuvering protocols are also evident, highlighted by the strong negative correlation (r = −0.81) between frontal obstacle proximity and linear velocity, which demonstrates appropriate deceleration in response to obstacles. Collectively, these quantified relationships affirm that the ANFIS controller successfully translates complex state information into coherent, goal-directed control actions.

**Figure 10:**
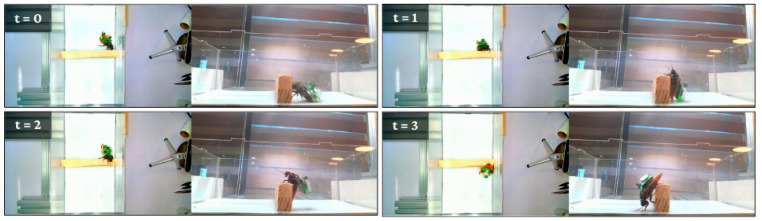
Unimpeded mobility of a *G. portentosa* biobot.

**Figure 11:**
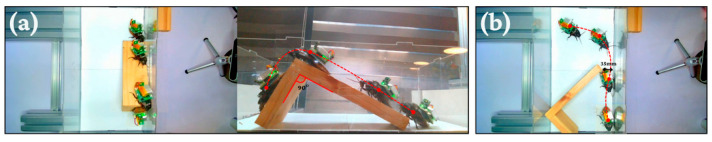
Locomotive performance on varied topographical challenges, (**a**) A time-lapse sequence shows the biobot successfully climbing over an A-shaped structure, requiring it to manage both ascent and descent. (**b**) The biobot makes a guided directional decision at a junction.

Figure 10 demonstrates the unimpeded mobility capabilities of a *G. portentosa* biobot navigating a vertical obstacle course. The sequence captures the cockroach’s natural climbing behavior while carrying an electronic backpack, showcasing the biobot’s ability to traverse complex three-dimensional terrain despite the additional payload. The roach exhibits characteristic exploratory movements, using its antennae to assess the obstacle before committing to the climbing motion. Throughout the ascent, the electronic interface remains securely attached without impeding the natural locomotor patterns, demonstrating the biocompatibility and mechanical stability of the backpack design. This mobility test validates the biobot’s potential for navigating confined spaces and vertical surfaces that would challenge conventional robotic platforms, highlighting the advantages of leveraging biological locomotion systems for specialized applications. Figure 11 illustrates the locomotive performance of *G. portentosa* biobots navigating varied topographical challenges in controlled experimental environments. Panel (a) presents a temporal sequence (t = 0 to t = 7) documenting the biobot’s successful traversal of an A-frame obstacle, demonstrating the organism’s innate ability to execute complex three-dimensional maneuvers including coordinated ascent and controlled descent phases. The red trajectory markers reveal the biobot’s path optimization as it negotiates the angular surfaces, showcasing how the biological locomotion system adapts to changing gravitational and mechanical constraints while maintaining the electronic payload’s stability. Panel (b) captures a critical behavioral milestone at t = 0 to t = 5, where the biobot encounters a Y-junction and executes a directional decision-making process. The tracked pathway indicates the organism’s natural exploratory behavior as it evaluates multiple route options before committing to a specific trajectory.

To assess robustness beyond nominal indoor trials, we evaluated two practical perturbations: light dust deposition and varied temperature/humidity. Using the same navigation protocols, we report direction MAE, response latency, and completion rate for each condition, providing an intermediate step toward field-like variability. The quantitative superiority of the ANFIS controller is comprehensively established through the metrics presented in Figure 14 and Table 6. The adaptive system demonstrated exceptional directional precision with a Mean Angular Error (MAE) of just 12.3° and a low Standard Deviation (S.D) of 3.7°, significantly outperforming the PID controller (MAE 27.8°, S.D 8.2°) and natural behavior (MAE 64.2°, S.D 15.1°). Responsiveness followed a similar trend, with ANFIS achieving the fastest response time at 0.4 s and the highest Consistency Index (C.I.) at 88%, compared to the sluggish 1.5 s response and 35% consistency observed in natural specimens. These capabilities translated directly into mission success: ANFIS-controlled specimens achieved an 81% maze completion rate with a mean time of 127 ± 31 s. In contrast, the PID controller achieved only a 37% success rate with significantly longer completion times (216 ± 48 s), while natural specimens struggled with a mere 22% success rate (274 ± 63 s), confirming the necessity of adaptive control for reliable navigation. During closed-loop operation, the controller uses lightweight, onboard state estimates derived from IMU and range sensors (e.g., heading/turn-rate and control errors) as its only real-time inputs. Trial videos are processed offline solely to extract reference trajectories and compute reported metrics; the video pipeline does not contribute to control decisions.

**Figure 12:**
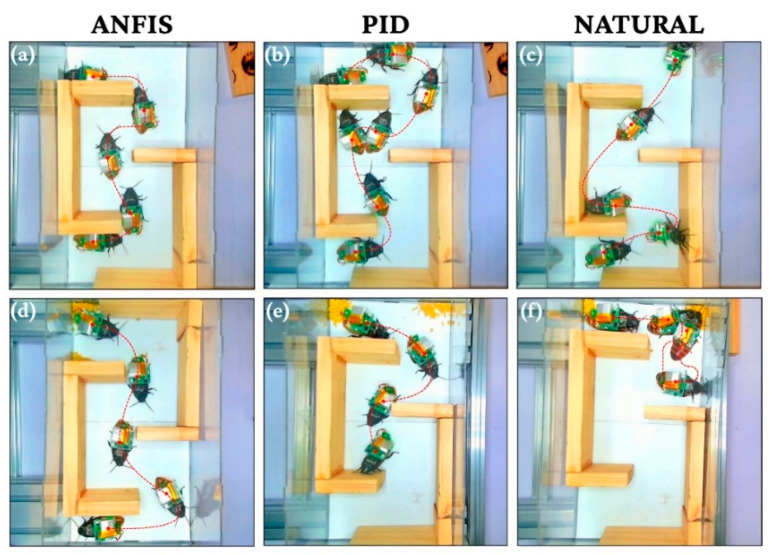
ANFIS, PID control and Natural behavior for lvl.1 maze-solving. (**a**–**c**) Maze-solving time-lapse without food; (**d**–**f**) Maze-solving time-lapse with food as a distractant.

**Figure 13:**
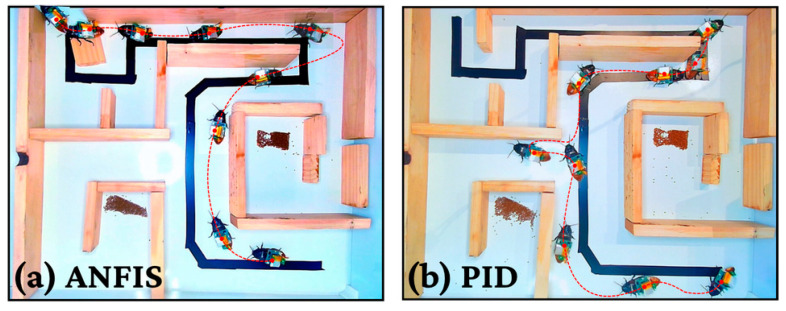
ANFIS (**a**) vs. PID (**b**) control for lvl.2 maze-solving.

Visual validation in the Level 1 maze (Figure 12) further illustrates these performance disparities under varying environmental conditions. Panels (a) and (d) confirm that the ANFIS-controlled biobot maintained a consistent, direct navigation path regardless of the presence of food distractants, demonstrating robust goal-directed behavior. Conversely, the PID controller (Panels b and e) showed visible inefficiency; while it achieved some guided navigation in neutral conditions, it failed to complete the maze when food was introduced, highlighting the inability of fixed-parameter systems to adapt to biological distractions. Natural behavior (Panels c and f) served as a baseline, showing erratic exploratory movements and significant delays caused by foraging instincts. Finally, in the complex, multi-turn Level 2 maze (Figure 13), the ANFIS controller demonstrated highly efficient path optimization, successfully negotiating intricate terrain where non-adaptive methods consistently failed, reinforcing the conclusion that adaptive neuro-fuzzy architectures are essential for robust bio-hybrid operation.

**Figure 14:**
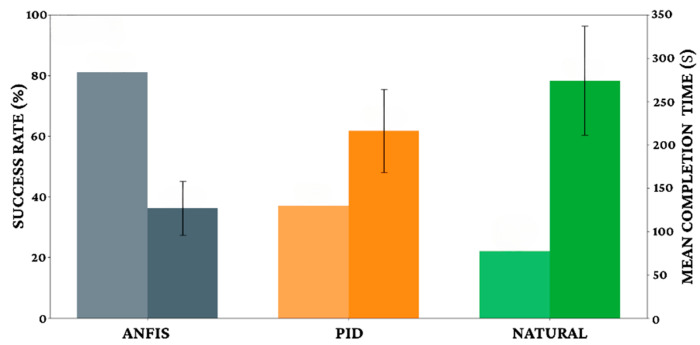
Maze navigation performance comparison.

Figure 14 and Table 6 collectively demonstrate the ANFIS controller’s superior precision and efficiency. The adaptive system achieved a Mean Angular Error (MAE) of 12.3° and a rapid response time of 0.4 s, significantly outperforming PID control (MAE 27.8°, 0.6 s) and natural behavior (MAE 64.2°, 1.5 s). These capabilities translated to an 81% maze completion rate with a mean time of 127 ± 31 s for ANFIS-controlled specimens, whereas PID achieved only 37% success (216 ± 48 s) and natural behavior just 22% (274 ± 63 s).

**Figure 15:**
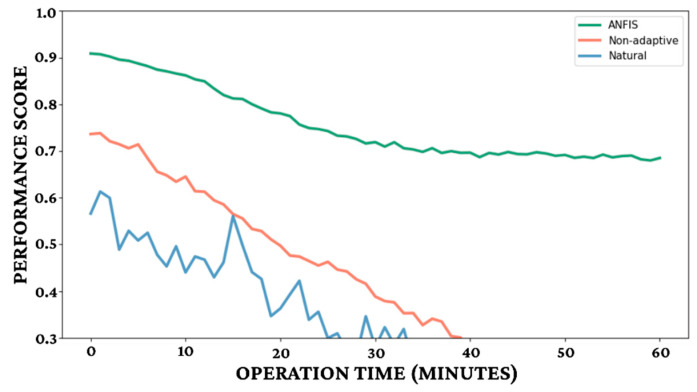
Temporal performance stability.

Figure 15 and Table 7 collectively demonstrate the critical long-term performance advantages of the ANFIS control system in bio-cybernetic cockroach navigation, Figure 15 visually illustrates the performance score of each control method over an operation time of 60 min. The ANFIS control consistently maintains a high performance score, starting at approximately 0.9 and stabilizing above 0.65 throughout the entire 60 min duration. In stark contrast, the non-adaptive controller, representing the PID, experiences a significant degradation in performance, starting lower and steadily declining to below 0.3 after approximately 40 min. The “Natural” behavior exhibits rapid and severe performance decay, becoming highly unstable within the first 20 min. Table 7 provides quantitative metrics that explain this superior temporal stability, particularly concerning habituation resistance. The ANFIS demonstrates exceptional resilience, boasting a response half-life of over 250 stimuli, a MRL of 82% of initial, a robust recovery rate (RR) of 79% per minute, and an effective control duration (ECD) of 47 min. Conversely, the PID control (non-adaptive) shows considerably lower habituation resistance, with a response half-life of approximately 68 stimuli, a MRL of only 26%, a RR of 13% per minute, and an ECD of just 26 min.

A pivotal finding of this research is the efficacy of the dual-timescale adaptation mechanism, which operates by rapidly adjusting consequent parameters for immediate responsiveness while slowly refining antecedent membership functions to learn long-term individual traits. This architecture allowed the system to build a personalized internal model for each specimen, treating habituation as a dynamic state variable (*Hab_Lvl*) rather than a fixed constraint. The result was a 3.5-fold increase in effective control duration compared to non-adaptive methods.

**Figure 16:**
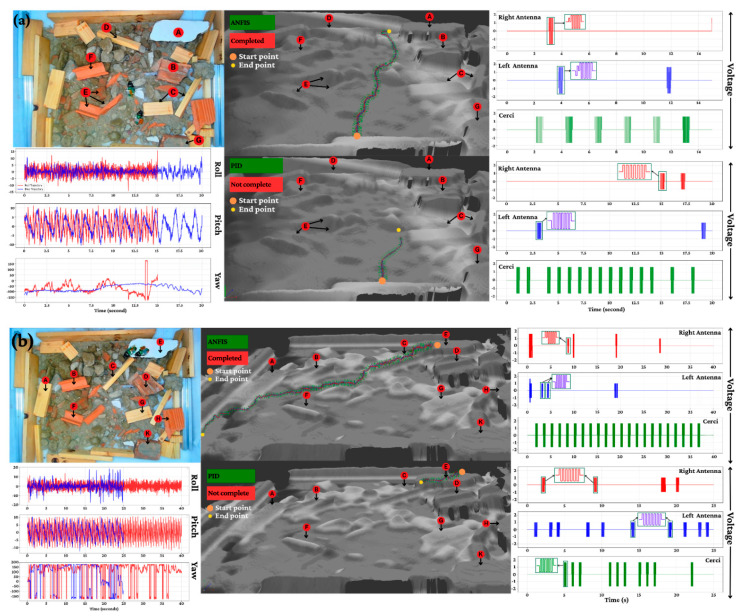
The best running of ANFIS and PID controllers in daytime scaled-down of experimental environment. (**a**) Go straight; (**b**) Go to target.

Figure 16a,b illustrate the operational efficacy in daytime rubble environments. The ANFIS controller achieved 100% mission completion for both straight-line and target-acquisition tasks. It demonstrated exceptional precision, maintaining a lateral deviation of less than 4 cm and a path efficiency exceeding 85%, with highly responsive pulse adjustments occurring within 0.3 s of obstacle detection. In stark contrast, the PID controller failed to complete these tasks, typically halting after traversing only 55–65% of the distance. It exhibited significant drift, with lateral deviations exceeding 18 cm and path efficiency falling below 50%, confirming its inability to handle complex terrain.

**Figure 17:**
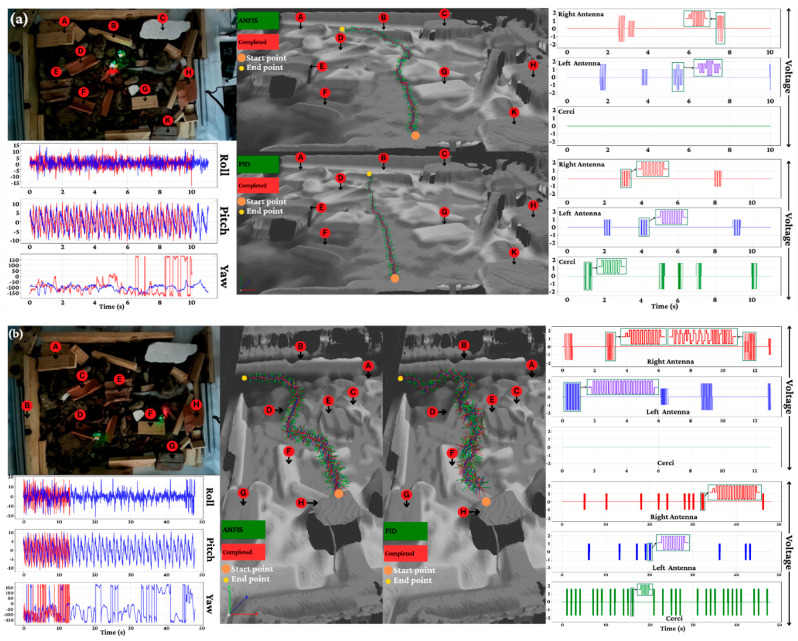
The best running of ANFIS and PID controllers in nighttime of scaled-down experimental environment. (**a**) Go straight; (**b**) Go to target.

In contrast, Figure 17 extends this evaluation to nighttime conditions, where visual cues are limited. The ANFIS controller maintained its robust performance, achieving 100% completion for straight-line navigation and 95% success in obstacle avoidance. Conversely, the PID controller struggled significantly, failing to reach targets and managing an obstacle clearance rate of only 40%. The physiological data further substantiates these behavioral results: ANFIS stimulation strategies resulted in less than 5% degradation in response intensity over the trial duration, whereas PID control showed a rapid decline in efficacy, with over 25% degradation observed in similar periods. These findings empirically validate that the ANFIS controller’s ability to dynamically modulate stimulation parameters provides a quantifiable and decisive advantage over conventional, non-adaptive control strategies in challenging, unstructured environments.

While the performance metrics are robust, it is crucial to acknowledge the limitations of the current implementation. The system, in its present form, still relies on an off-board workstation for the computationally intensive tasks of video processing and state estimation. This tether to external processing infrastructure, while necessary for this proof-of-concept stage, precludes true autonomy in real-world environments. Furthermore, all trials were conducted within a controlled laboratory setting. A rule-cap ablation shows diminishing returns beyond ∼100 rules; we adopt ≈105 as the accuracy–cost knee point (Table 8).

The complexities of a real-world disaster zone—with unpredictable terrain, dynamic obstacles, and variable lighting—would present a far greater challenge than the structured arenas used in our experiments. These limitations, however, illuminate a clear and exciting path for future research. The primary technological hurdle to overcome is the migration of the entire control and perception pipeline onto the onboard system. Advances in low-power, high-performance microcontrollers and neuromorphic processing units may soon make fully autonomous, untethered biobots a reality. Future work must also focus on validating these systems in progressively more complex and unstructured environments to test the true limits of their adaptability. Finally, the behavioral repertoire could be expanded significantly. Future iterations could integrate additional sensors (e.g., chemical or thermal) and train the ANFIS to perform more sophisticated tasks, such as source localization, pattern recognition, or even collaborative, swarm-based exploration. Environmental perturbations produce modest, bounded degradation. The dual-timescale adaptation helps preserve performance by quickly adjusting consequents while slowly re-tuning membership functions under sustained drift; simple sealing/dust shielding and lightweight self-check routines are recommended for deployment.

## 5. Conclusions

In conclusion, this investigation demonstrates that the fusion of adaptive neuro-fuzzy control with a living biological system represents a paradigm shift in bio-cybernetics. The ANFIS controller significantly surpasses conventional methodologies, creating a more effective, resilient, and enduring bio-hybrid platform. This work lays a critical foundation and offers a clear roadmap for the next generation of bio-cybernetic systems, underscoring the importance of integrating advanced computational intelligence with responsible and ethical research practices to unlock their full potential.

## Figures and Tables

**Table 1:** Experimental Parameters and Key Findings.

Parameter	Range/Value	Key Findings
Specimens	24 individuals	Response thresholds varied by 45% between individuals
Stimulation Frequency	10–200 Hz	Optimal response range: 20–80 Hz
Inter-trial Intervals	≥3 min	Prevented acute habituation during testing
Short-term Habituation	6–12 episodes	65% recovery after 5 min, complete after 15 min
Long-term Adaptation	After 45 min	Progressive threshold elevation despite rest intervals

**Table 2:** Qualitative comparison of nonlinear controllers for habituation-aware insect bio-bot control.

Method	Main Advantage	Key Limitation in Bio-Bots	Compute & Data Demand	Best-Fit Scenario
ANFIS	Interpretable + online adaptation; efficient inference	Rule-base design/dimensionality growth if uncontrolled	Low–moderate compute; low data (online)	Real-time, resource-limited control with individual drift
Sliding Mode Control	Robust to bounded uncertainty; simple online computation	Chattering/smoothing complexity; needs model bounds	Low compute; low data	Systems with reliable bounds and strong robustness needs
Deep RL	High expressive power for complex nonlinearities	Sample-hungry; safety/exploration concerns; training overhead	High compute; high data	When safe training + simulators + offline training are feasible

**Table 3:** ANFIS Model Configuration Summary.

Parameter	Configuration	Key Findings
Input	8	Response thresholds varied by 45% between individuals
Output	3	Stimulus amplitude (V), frequency (Hz), duration (ms)
MF Type	gbellmf	Optimal for smooth nonlinear approximation with adjustable parameters
MF per Input	3 to 5	Adaptive based on input variable complexity and range
Fuzzy Rule Type	Interval Type-1 Fuzzy	Linear output consequents enable efficient parameter optimization
Number of Fuzzy Rules	80 to 150	Dynamic rule generation balancing expressiveness with computational efficiency
Antecedent Operator	Algebraic Product	Smooth differentiable operation for gradient-based learning
Implication Method	Product for ANFIS	Maintains differentiability for backpropagation-based parameter updates
Aggregation Method	Algebraic Sum	To combine outputs from all contributing rules
Defuzzification	Weighted Average	Computationally efficient for real-time control applications
Primary Learning	Hybrid Learning	Combines GD and LSE
Premise Parameters	GD with Momentum	Learning rate: 0.01, momentum coefficient: 0.9
Consequent Parameters	RLS	Forgetting factor: 0.995 for adaptive tracking
Training Epochs	250	Sufficient for convergence while preventing overfitting
Online Adaptation	Enabled	Real-time parameter updates for habituation compensation and individual adaptation
Validation Method	Cross-validation (k = 5)	Ensures generalization capability across different specimens
Convergence Criterion	After 45 min	Training termination when root mean square error falls below threshold

**Table 4:** Input variables for the ANFIS controller.

Parameter	Configuration	Range	Fuzzy Sets
Prox_F	Proximity—Front	[0; 1]	{PF_Far, PF_Medium, PF_Near, PF_VeryNear}
Prox_B	Proximity—Back	[0; 1]	{PB_Far, PB_Medium, PB_Near, PB_VeryNear}
Vel_Lin	Current Linear Velocity	[0; 1]	{VL_Stop, VL_Slow, VL_Medium, VL_Fast}
Vel_Ang	Current Angular Velocity	[−1; +1]	{VA_FastLeft, VA_SlowLeft, VA_Straight, VA_SlowRight, VA_FastRight}
Dist_Targ	Distance to Target	[0; 1]	{DT_VeryClose, DT_Close, DT_Medium, DT_Far}
Err_Bear	Target Bearing Error	[−180; 180]	{EB_HardLeft, EB_Left, EB_Ahead, EB_Right, EB_HardRight}
Accel_Ang	Angular Velocity	[−1; +1]	{AA_Decel, AA_Steady, AA_Accel}
Hab_Lvl	Estimated Habituation Level	[0; 1]	{HL_Low, HL_Medium, HL_High}

**Table 5:** Membership Function Parameter Optimization.

Variable Name	Membership Function (MF)	Initial Center Value(s)	Initial Width Value(s)	Optimized Center Value(s)	Optimized Width Value(s)	Percent Change
Prox_F	PF_Far	0.15	0.10	0.17	0.09	+13.33%
	PF_Medium	0.40	0.10	0.38	0.11	−5.00%
	PF_Near	0.65	0.10	0.62	0.09	−4.62%
	PF_VeryNear	0.90	0.10	0.88	0.08	−2.22%
Prox_B	PB_Far	0.15	0.10	0.16	0.10	+6.67%
	PB_Medium	0.40	0.10	0.41	0.09	+2.5%
	PB_Near	0.65	0.10	0.63	0.10	−3.08%
	PB_VeryNear	0.90	0.10	0.89	0.09	−1.11%
Vel_Lin	VL_Stop	0.05	0.05	0.04	0.04	−20.00%
	VL_Slow	0.30	0.15	0.28	0.13	−6.67%
	VL_Medium	0.60	0.15	0.63	0.14	+5.00%
	VL_Fast	0.90	0.10	0.92	0.09	+2.22%
Vel_Ang	VA_FastLeft	−0.80	0.20	−0.85	0.18	+6.25%
	VA_SlowLeft	−0.30	0.20	−0.25	0.17	−16.67%
	VA_Straight	0.00	0.15	0.00	0.13	0.00%
	VA_SlowRight	+0.30	0.20	+0.25	0.17	−16.67%
	VA_FastRight	+0.80	0.20	+0.85	0.18	+6.25%
Dist_Targ	DT_VeryClose	0.10	0.10	0.08	0.08	−20.00%
	DT_Close	0.30	0.15	0.27	0.13	−10.00%
	DT_Medium	0.60	0.20	0.62	0.18	+3.33%
	DT_Far	0.90	0.10	0.93	0.09	+3.33%
Err_Bear	EB_HardLeft	−0.80	0.20	−0.85	0.18	+6.25%
	EB_Left	−0.40	0.20	−0.35	0.17	−12.50%
	EB_Ahead	0.00	0.20	0.00	0.15	0.00%
	EB_Right	+0.40	0.20	+0.35	0.17	−12.50%
	EB_HardRight	+0.80	0.20	+0.85	0.18	+6.25%
Accel_Ang	AA_Decel	−0.50	0.25	−0.55	0.22	+10.00%
	AA_Steady	0.00	0.20	0.00	0.18	0.00%
	AA_Accel	+0.50	0.25	+0.55	0.22	10.00%
Hab_Lvl	HL_Low	0.20	0.15	0.18	0.14	−10.00%
	HL_Medium	0.50	0.15	0.52	0.13	+4.00%
	HL_High	0.80	0.15	0.85	0.12	+6.25%

**Table 6:** Directional control performance metrics.

Control Method	MAE (°)	S.D (°)	Response (s)	C.I (%)
ANFIS	12.3	3.7	0.4	88
PID	27.8	8.2	0.6	65
Natural	64.2	15.1	1.5	35

**Table 7:** Habituation Resistance Metrics.

Control Method	Response Half-Life	MRL (% of Initial)	RR (%/min)	ECD (min)
ANFIS	>250	82%	79%	47
PID	~68	26%	13%	26

**Table 8:** Ablation study on the maximum number of active fuzzy rules.

Max Active Rules	Direction MAE (°) ↓	Response Time (s) ↓	Completion Rate (%) ↑	Relative Inference Cost ↑
45	17.8	0.41	72	1.0×
75	14.2	0.41	78	1.5×
105	12.3	0.40	81	2.1×
135	12.1	0.40	81	2.7×

## Data Availability

The data have already been included in this paper.
